# Predictors of serum concentrations of polybrominated flame retardants among healthy pregnant women in an urban environment: a cross-sectional study

**DOI:** 10.1186/1476-069X-12-23

**Published:** 2013-03-08

**Authors:** Megan K Horton, Sabine Bousleiman, Richard Jones, Andreas Sjodin, Xinhua Liu, Robin Whyatt, Ronald Wapner, Pam Factor-Litvak

**Affiliations:** 1 Department of Epidemiology, Mailman School of Public Health, Columbia University, New York, NY, USA; 2 Sergievsky Center, Columbia University, New York, NY, USA; 3 Department of Obstetrics and Gynecology, Columbia University Medical Center, New York, NY, USA; 4 Centers for Disease Control and Prevention, Atlanta, GA, USA; 5 Department of Biostatistics, Mailman School of Public Health, Columbia University, New York, NY, USA; 6 Columbia Center for Children’s Environmental Health, Department of Environmental Health Sciences, Mailman School of Public Health, Columbia University, New York, NY, USA; 7 722 W. 168th St, Room 735, New York, NY, 10033, USA

**Keywords:** PBDEs, Human exposure, Diet, Lifestyle, Pregnancy

## Abstract

**Background:**

Polybrominated diphenyl ethers (PBDEs) are a class of brominated flame retardants commonly used in a wide range of products. Prenatal exposure to PBDEs has been associated with adverse neurodevelopment. Our objective was to characterize predictors of exposure to PBDEs among a multi-ethnic, low-income cohort of pregnant women enrolled from highly urban communities in New York City between years 2009–2010.

**Methods:**

During the first half of pregnancy we collected 316 maternal serum samples and administered an extensive questionnaire including items on demographics, diet and lifestyle. We measured 12 PBDE congeners in blood samples. Using bivariate and multivariate approaches, we regressed the most commonly detected PBDE congeners (PBDE-47, -99, -100 and -153) against potential demographic, dietary and lifestyle predictor variables.

**Results:**

At least one PBDE congener was detected in each serum sample. Our analyses demonstrate unique predictor patterns for PBDE-47, -99, -100 and -153 based on demographic, lifestyle and dietary characteristics of women. Higher education and increased use of household electronics were associated with higher levels of all 4 congeners. Six characteristics were associated with PBDE-153 serum concentrations, more than for any other congener. These include maternal education, household income, body mass index, solid dairy consumption, processed meat consumption and frequent use of household electronics.

**Conclusions:**

PBDE exposure in this widespread in this cohort, though levels are lower than previous assessments of US pregnant women. Lower levels may be in response to legislation restricting the production, sale and use of these compounds. In our cohort, we did not observe any individual predictor or a consistent pattern of several predictors representing a significant source of PBDE exposure. These data suggest that legislation and policy may be more effective at reducing exposure than personal lifestyle modifications.

## Background

Since the 1970’s, polybrominated diphenyl ethers (PBDEs) have been used as flame retardants in consumer products such as plastics, electronics, textiles and construction materials. Like other persistent organic pollutants (POPs) such as polychlorinated biphenyls (PCBs) and dichlorodiphenyltrichloroethane (DDT), PBDEs are persistent, lipid soluble and bioaccumulative [1]. Decades of widespread use led to ubiquitous environmental contamination and human exposure [2]. A 2008 biomonitoring study detected PBDEs in 97% of blood samples collected from a representative sample of residents of the United States [3]. Due to growing environmental and human health concerns, considerable legislation has addressed the production, sale and use of these compounds [4,5]. Despite regulatory restrictions, sources of PBDEs remain and continue to contaminate the environment resulting in considerable human exposure.

Humans are exposed to PBDEs via several sources including diet and consumer products. The relative contribution of each source to overall body burden has not been well established in pharmacokinetic models [6]. Because they are persistent and bioaccumulative, food items including fish from high trophic levels and high fat dairy products contain elevated concentrations of PBDEs [7]. The presence of PBDEs in various everyday household products such as electronics, carpeting, and upholstery may also lead to considerable exposure. Non-covalent binding of PBDEs to the surface of consumer items allows them to readily leach into household dust [8]. Household dust represents an aggregate of indoor source of PBDEs [9-12]. Recent estimates suggest more than 80% of PBDE exposure is from unintentional ingestion of contaminated house dust [13]. Neither dermal absorption or inhalation of PBDEs are considered significant contributions to PBDE body burden [14-16].

Recent studies suggest PBDE exposures are not homogenous across diverse racial and ethnic groups and that non-white populations may face higher exposures to PBDEs than white populations (reviewed in [17]). Though several studies have examined racial and ethnic differences in PBDE body burden, the factors associated with these disproportionate exposures are not yet understood.

While several studies have explored individual determinants of PBDE body burdens in the general public, here we are particularly interested in characterizing predictors of exposure to PBDEs in pregnant women. PBDE toxicity is largely thought to be due to endocrine disruption [18], particularly of thyroid hormone function. Pregnant women may be more vulnerable to thyroid disruption as pregnancy represents a period of increased demand on the maternal thyroid gland. Developmental toxicity may also result from direct action of PBDEs on the developing brain. PBDEs readily cross the blood placenta barrier [19], and the blood–brain barrier, accumulating in the central nervous system [20]. PBDEs or their metabolites have been shown to directly affect aspects of brain development including neuronal proliferation, migration, synaptogenesis, synaptic plasticity and myelination [21]. Because the fetal period represents a critical window of vulnerability to the adverse effects of PBDEs, it is important to identify and characterize sources of exposure to PBDEs in women of childbearing age to identify vulnerable subpopulations and inform intervention studies aiming to reduce exposure.

The principle objective of the current study was to characterize the extent of maternal exposure to PBDEs among predominately Hispanic pregnant women living in New York City and investigate demographic, lifestyle, and dietary characteristics to better characterize predictors of exposure. This cross sectional study examined the relationship between maternal serum concentrations of 4 PBDE congeners and demographic, dietary and lifestyle characteristics among 316 healthy pregnant women living in New York City.

## Methods

### Study design

The study sample comprises mother-child pairs participating in the Endocrine Disruption in Pregnant Women: Thyroid Disruption and Infant Development Study. The cohort consists of 316 healthy pregnant women ages 16–35 enrolled from two New York City prenatal clinics between September 2009 and December 2010. All women presenting at prenatal clinics for care before approximately 20 weeks gestational age (based on reported date of last menstrual period and the earliest ultrasound) without a known multifetal gestation were eligible for enrollment. Subjects were excluded if they had medical complications (including chronic hypertension, diabetes, or epilepsy), or were known to abuse illicit drugs and/or alcohol. Baseline data was collected on all mothers screened at the prenatal clinics and included demographic characteristics, measures of social circumstances (marital status, home ownership, income, education, payment type for medical care), number of previous pregnancies, infections and substance use (tobacco, alcohol and street drugs) during the current pregnancy, and pre-pregnancy weight and height. The Institutional Review Board (IRB) of Columbia University approved this study protocol. Written informed consent was obtained from all subjects and included a statement that all data presented for publication would be grouped, rather than individual. The evaluation by the Centers for Disease Control and Prevention (CDC, Atlanta, GA, USA) determined that their role in this did not include human subjects research.

### Biological sample collection and PBDE exposure

Maternal blood (10 mL) was collected during the first half of pregnancy at a regularly scheduled prenatal blood draw. Blood was collected in silicon-coated (PBDE-free) vacutainers provided by the CDC to avoid contamination. Aliquots of 2 ml serum from each subject were sent to the CDC for measurement of PBDEs and serum lipids (total triglycerides and cholesterol). Serum PBDEs were measured using isotope dilution high resolution mass spectrometry on a MAT95XP (ThermoFinnegan MAT; Bremen, Germany) instrument. Serum lipids were measured via commercially available test kits from Roche Diagnostics Corp. (Indianapolis, IN). Details of the analytical methodology for measuring PBDEs and lipids in sera, including quality control, reproducibility and limits of detection are provided elsewhere [22]. Serum samples were available for all 316 participants. However, the number of individual serum PBDE congeners available for analyses varied as a less than 1% of samples did not pass quality control/quality assurance (QA/QC) procedures and the concentrations were not reportable.

### Exposure questionnaire data

Based on a comprehensive literature review and a questionnaire developed at the University of British Columbia (Glenys Webster, personal communication), we developed a structured questionnaire to ascertain determinants of PBDE exposure from the year prior to conception through the time of interview. Questions regarding dietary intake focused specifically on the foods consumed during the 6 months prior to conception and throughout pregnancy. A trained research nurse administered the 45-minute questionnaire to each participant in the study during a routine clinic visit. We queried potential dietary and non-dietary (lifestyle) predictors. Based on prior publications on the foods that most contribute to PBDE intake [8], we focused our dietary analysis on 14 food items included in the following groups: dairy, fish, beef, pork, chicken and fast food. For lifestyle characteristics, we focused on 5 main predictors: current employment, use of a foam chair, reporting of exposed foam in the home, carpeting, and number of household electronics.

For each dietary category, each participant was asked if she consumed a particular food item (yes/no) and the frequency of consuming one serving size of the food item (ranging from < 1 time/week to > 7 times/week) throughout pregnancy. Serving size estimates were adapted from United States Department of Agriculture (USDA) resources. Food items were examined a) individually as the frequency of consuming one serving size and as b) groups of foods generated by summing the frequencies of consuming one serving size of all items in a group (i.e. total dairy items). Dairy foods included milk, type of milk (skim, 1%, 2%, whole or soy), cheese, butter, cottage cheese, ice cream/frozen yogurt, and cream cheese. The association between dairy foods and serum PBDE levels was examined for individual dairy items and for total dairy (sum of the frequencies of consuming one serving size of any dairy item), liquid dairy (sum of the frequencies of consuming one serving size of milk and/or ice cream) and solid dairy (sum of the frequency of consuming one serving size cheese, yogurt, cottage cheese, butter and/or cream cheese). Participants were asked about meat consumption (beef, pork, chicken and/or turkey, sausage (beef or pork), bacon, hotdogs, and other meat). Meat items were evaluated individually. Additionally, processed meats including sausage, bacon and/or hotdogs were summed and treated as one variable. Fish items included tuna, salmon (fresh farmed, fresh wild, canned), other fatty fish, other white fish, crab, lobster, shrimp, mussels, oysters, scallops, and other shellfish. The association between fish items and serum PBDE levels was examined individually for salmon (all types) and other fatty fish, tuna, and any shellfish (sum of the frequency of consumption of any shellfish). Participants were also queried about consumption of processed food items including microwave popcorn, movie theater popcorn, pizza (delivered in a box), take-out food, and prepackaged food prepared in the microwave. Processed food items were examined individually and as a sum of the frequency of consumption of any of the five items. If a participant reported consumption of organic foods, she was asked which categories of food were organic (vegetables and/or fruits, meat, dairy products, or other items) and how often she consumed them (always, most of the time, some of the time, never). Finally, participants were asked about consumption of hot beverages including caffeinated coffee, decaffeinated coffee, caffeinated tea, decaffeinated tea and other hot beverages. Questions included the frequency of consumption as the number of cups as well as whether the cups were paper or plastic disposable cups.

We scaled each food item response into the number of times per week a serving size of a food item was consumed, examined the distributions, and modeled 3-level categorical variables to reflect no/low, medium and high patterns of consumption. Excepting consumption of solid dairy foods and processed meat, the ‘no/low’ level reflects reported consumption of the item less than or equal to 5 times in one week. The ‘medium’ level reflects reported consumption of the item between 6 and 10 times in one week. ‘High’ consumption reflects greater than 10 times in one week. We selected these cut-points in an attempt to develop a categorical variable with three equally sized levels. For solid dairy foods and processed meat, consumption was dichotomized to <5 times per week compared to ≥ 5 times per week; for these categories a third category was created for missing data. 35 subjects were missing solid dairy consumption data and 62 subjects were missing processed meat consumption data.

Lifestyle questions included occupation, type and location of residence, electronic items in the home and transportation. Participants were asked if they worked outside the home during the year prior to or during the current pregnancy. If yes, the type of job was reported along with the length of time at the job, types of materials involved (plastics, electronics, plastic or foam). Participants reported whether the job involved working on a computer or at a copy machine. Questions about hobbies included the type of hobby and whether any hobbies included working with foam, plastics, or heavy fabric. Residential history included city, zip code and duration of time at current residence. Participants were asked if the home contained any damaged or crumbling furniture or whether they frequented a home or building with exposed or crumbling foam furniture. We also asked participants if they customarily covered sofas, mattresses, upholstered chairs, or other household furniture in plastic. Questions about bedding included the type of primary and secondary (if applicable) bedding (mattress, futon or sofa) and pillow (foam or down). We asked which of the rooms, if any, were carpeted. With respect to electric appliances, we asked if the participants had any of the following items in their home and if so how many and duration of daily use: televisions, computers, printers/fax machine/scanner/copiers, stereo equipment, clock radio, microwave, toaster/toaster oven, electric coffee maker, or bread maker. We dichotomized the number of electric appliances in the home to < 10 items compared to ≥ 10 items. Finally, participants were asked if they drove in or regularly rode in a car with ripped seats or exposed foam and if so, how many hours each week were spent in the car.

### Data analysis

Only PBDEs with ≥ 50% detectable levels (PBDE-47, -99, -100 and -153) were further evaluated in this paper. These PBDE congeners had skewed distributions. Medians and interquartile ranges are presented for PBDEs concentration per gram lipid (ng/g serum). Decile categories of PBDE exposure were calculated according to the distribution of values in the dataset. We used the Kruskal Wallis one-way analysis of variance by ranks to evaluate the univariate relationships between PBDE congeners and sample characteristics.

We used regression models to estimate the associations between potential predictors and PBDE concentrations. PBDE concentrations below the limit of detection (LOD) were treated as left censored observations [23]. We ran regression models with several different distributions and selected the best-fitting distribution for each PBDE congener, based on the log likelihood. The general gamma distribution fitted PBDE-47 and -153 and the log logistic distribution fitted PBDE-99 and -100 better than other choices (e.g. Weibull). Consequently, gamma and/or log logistic regression models were used, as appropriate, to estimate the associations between predictors and each PBDE congener. First, we used single predictor models to determine if any single demographic, dietary or lifestyle factor was a significant predictor of an individual PBDE congener outcome at p < 0.10. Second, we extended the model for each outcome to include all predictors that were significant at p < 0.10. The final models were estimated including all predictors that remained significant at p < 0.10. In these models the estimated coefficient of predictor variables is interpreted as the ratio of means in the outcome variable comparing either unit changes (for continuous variables) or categories compared to the reference category (for categorical variables), adjusted for other covariates [24]. The generalized coefficient of determination R^2^ was used to indicate the proportion of variability in the data that is accounted for by the statistical model with multiple predictors [25].

All statistical analyses were conducted using PAWS SPSS 18.0 (IBM, Armonk, New York) or SAS 9.2 (SAS Institute Inc, Cary, North Carolina).

## Results

### Population characteristics

Socio-demographic characteristics of the mothers participating in this cohort are presented in Table 1. This is a multi-ethnic cohort consisting of Hispanic (67%), non-Hispanic White (27%) and African American (6%) women. The mean maternal age was 29 (range 16–43) years. The majority of women (84%) completed high school and obtained some college education at the time of enrollment. Fifty women (16%) had obtained graduate degrees. 63% reported an annual family income < $25,000. Most women (70%) were multiparous; the median number of previous pregnancies was 1.

**Table 1:** Selected demographics for the cohort consisting of 316 pregnant women enrolled between September 2009 and December 2010 from clinics in the New York City area

**Variable**	N (%)*
**Race/ethnicity**	
African American	19 (6%)
Non Hispanic White	84 (27%)
Hispanic	211 (67%)
**Maternal age (years, range)**	29 (16 to 43)
**Maternal education**	
Less than high school diploma	50 (16%)
High school or equivalent	75 (24%)
College	128 (44%)
Graduate	50 (16%)
**Prepregnancy BMI**	
Underweight (< 18.5)	19 (6%)
Normal (18.5 to 24.9)	169 (55%)
Overweight (25–29.9)	78 (25%)
Obese (> 30)	42 (14$)
**Household income**	
<$25,000	198 (63%)
$25,000 – 50,000	34 (10%)
>$50,000	84 (27%)
**Multiparous**	220 (70%)

*Missing values for predictors: race/ethnicity (2), maternal education (3), prepregnancy BMI (8), parity (1).

### PBDE concentrations in maternal serum

Lipid adjusted concentrations of all PBDE congeners analyzed are presented in Table 2. All of the 316 maternal serum samples contained at least one detectable PBDE congener. As shown in Table 2, for most congeners (PBDE-17, -18, -66, -85, -154, -183, -209) the majority of concentrations were below the LOD. These congeners were not included in the following analysis.

**Table 2:** **Lipid adjusted concentrations and congener distributions of PBDE in maternal serum** (**n** = **316**)

**Congener**	**N**	**% > LOD**	**Median LOD ng/g serum lipid)**^**a**^	**Median (ng**/**g serum lipid)**^**b**^	**Range (ng/g serum lipid)**	**Congener distribution(median %)**^**c**^
PBDE-17	298	6.7	0.50	0.35	ND-1.8	1.69
PBDE-28	316	45.6	0.50	0.42	ND-63.7	2.03
PBDE-47	316	99.1	1.20	7.90	ND-1600	38.15
PBDE-66	316	3.2	0.50	0.35	ND-2.5	1.69
PBDE-85	316	24.7	0.70	0.35	ND-38.3	1.69
PBDE-99	316	83.5	0.50	1.60	ND-377	7.73
PBDE-100	316	91.1	0.50	1.70	ND-301	8.21
PBDE-153	315	98.4	0.50	2.95	ND-206	14.24
PBDE-154	316	14.6	0.50	0.35	ND-34.1	1.69
PBDE-183	316	15.5	0.50	0.35	ND-2.9	1.69
PBDE-209	311	19.3	5.40	4.04	ND-38.2	19.51
PBB-153	314	38.9	0.50	0.35	ND-84.3	1.69

ND = non detect. The concentration of PBDE measured was less than the limit of detection (LOD).

^a^Median LOD. LODs differed between individuals due to the amount of sample collected provided for analysis, sample quantity ranged from 0.374-2.002 g, mean ± se = 1.898 ± 0.009.

^b^For samples with PBDE concentrations < LOD, the value of LOD/sqrt 2 was substituted.

^c^PBDE congener distribution (median %) calculated as the percent contribution of the sum of 12 congeners to the total sum of median congener concentrations (ΣPBDE).

**Figure 1:**
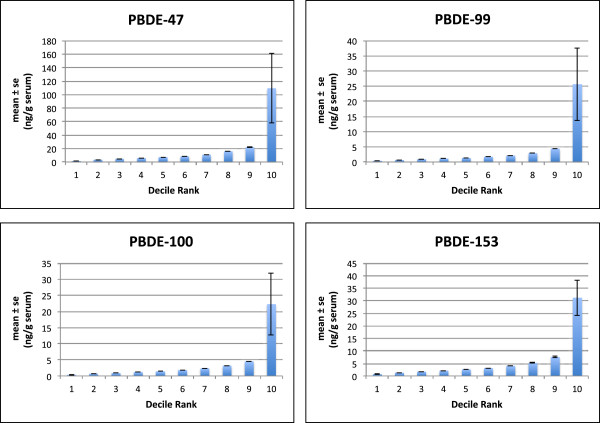
**Concentrations of PBDE-47, -99, -100 and -153 measured in 316 maternal serum samples (mean ± se ng/g serum) presented as deciles of exposure (10 groups of equal frequency) revealing negatively skewed distributions of exposure. **For each of the 4 congeners presented, over 90% of subjects have relatively low PBDE concentrations (PBDE-47 < 26.9 ng/g lipid, PBDE-99 < 5.60 ng/g lipid, PBDE-100 < 5.60 ng/g lipid and PBDE-153 < 10.10 ng/g lipid) Ranges for the highest 10% of PBDE serum concentrations are as follows; PBDE-47 (27.7-1600.0 ng/g lipid), PBDE-99 (5.60-377.0 ng/g lipid), PBDE-100 (5.60-301.0 ng/g lipid), PBDE-153 (10.10-206.0 ng/g lipid).

PBDE-47, PBDE-99, PBDE-100 and PBDE-153 had detection frequencies greater than 80%. PBDE-47, -99 and -100 were most highly correlated with each other (Spearman’s r > 0.85, p < 0.001). PBDE-153 was not as highly correlated with other congeners (Spearman’s r < 0.60, p < 0.001). As demonstrated in Figure 1 representing the decile categories of PBDE concentrations, PBDE concentrations for all 4 frequently detected congeners were skewed to the left. For each of the 4 congeners presented, over 90% of subjects have relatively low PBDE concentrations and 10% have concentrations 10–20 fold higher than the median.

### Predictors of PBDE concentrations in maternal serum

Table 3 compares median and interquartile ranges of lipid-adjusted PBDE concentrations by demographic, dietary and lifestyle characteristics. Based on these univariate analyses, four characteristics were associated with higher maternal PBDE-47 concentrations. Subjects reporting a high school or equivalent education at the time of enrollment had higher PBDE-47 levels than subjects with more (college or graduate school) or less education. Also, consumption of processed meat (compared to no consumption), reporting of ≥ 10 household electronics (compared to < 10) and current work (compared to unemployed) were associated with higher PBDE-47 levels. For PBDE-99, reporting ≥ 10 household electronics was positively associated with maternal serum concentrations. For PBDE-100, both processed meat consumption and reporting of ≥ 10 household electronics were associated with higher maternal serum concentrations. Median PBDE-153 levels were highest among Non Hispanic Whites and lowest among Hispanics. Though race/ethnicity did not significantly predict levels of PBDE-47, -99, or -100, it appeared levels of these congeners were highest among African Americans and lowest among Non Hispanic Whites and Hispanics. Also different than PBDE-47, PBDE-153 levels were highest among college graduates compared to all other education categories. PBDE-153 levels were higher among subjects reporting an annual income > 50K and subjects with normal prepregnancy body weight. Higher consumption of solid dairy products and consumption of any processed meat was associated with higher PBDE-153 levels. With respect to lifestyle characteristics, having carpet in the home and current employment were associated with higher PBDE-153 levels.

**Table 3:** **Median and interquartile ranges of lipid**-**adjusted PBDE concentrations **(**ng**/**g lipid**)^**b **^**in maternal blood collected upon delivery by demographic**, **dietary and lifestyle characteristics**

**Characteristics/Predictors**		**PBDE 47**	**PBDE 99**	**PBDE 100**	**PBDE 153**^**a**^
		**Median (IQR)**	**Median (IQR)**	**Median (IQR)**	**Median (IQR)**
**Demographic**					
	**Race**/**Ethnicity**				
	African American	9.20 (19.8)	1.70 (3.9)	1.90 (2.6)	3.00 (2.1)
	Non Hispanic White	7.80 (9.7)	1.45 (1.9)	1.70 (2.3)	3.40 (6.7)
	Hispanic	7.70 (11.6)	1.60 (2.1)	1.60 (2.4)	**2**.**80 **(**2**.**9**)*
	**Education**				
	Less than high school	7.10 (11.6)	1.20 (2.2)	1.45 (2.3)	2.15 (2.8)
	High school or high school equivalent	**9**.**00 **(**14**.**0**)	1.80 (3.1)	2.00 (2.7)	2.70 (2.7)
	College	8.20 (12.0)	1.60 (1.8)	1.60 (2.1)	**3**.**20 **(**3**.**6**)
	Graduate School	7.10 (7.1)*	1.40 (1.9)	1.65 (2.1)	3.00 (2.7)*
	**Income**				
	< 25	7.70 (11.5)	1.60 (1.8)	1.60 (2.5)	2.60 (2.9)
	25-50K	9.00 (12.5)	1.80 (3.1)	2.15 (2.3)	3.20 (3.3)
	> 50 K	7.90 (10.9)	1.40 (2.0)	1.55 (2.1)	**3**.**60 **(**5**.**5**)*
	**BMI**				
	Underweight	7.80 (4.4)	1.54 (1.6)	1.40 (1.3)	2.90 (2.8)
	Normal	7.90 (12.2)	1.60 (1.9)	1.60 (2.6)	**3**.**20 **(**3**.**9**)
	Overweight	8.45 (11.7)	1.61 (2.2)	2.05 (2.0)	2.90 (3.3)
	Obese	7.60 (10.4)	1.30 (1.5)	1.70 (2.2)	2.15 (1.9)*
**Dietary**					
	**Solid Dairy **(**servings**/**week**)				
	< 5	7.30 (10.4)	1.40 (2.0)	1.50 (2.1)	2.30 (2.4)
	≥ 5	7.80 (10.9)	1.60 (1.8)	1.60 (2.2)	**3**.**10 **(**3**.**5**)*
	**Processed meat **(**any**)				
	No	7.00 (6.6)	1.40 (1.6)	1.40 (1.4)	2.35 (2.1)
	Yes	**8**.**90 **(**15**.**6**)*	1.70 (2.9)	**2**.**00 **(**3**.**3**)**	**3**.**90 **(**4**.**7**)*
**Lifestyle**					
	**Electronics (# of household electronics)**				
	< 10	7.00 (9.6)	1.40 (1.9)	1.40 (2.0)	2.80 (3.5)
	≥ 10	**9**.**40 **(**11**.**5**)*	**1**.**90 **(**2**.**5**)**	**2**.**20 **(**2**.**6**)**	**3**.**20 **(**4**.**5**)*
	**Carpet**				
	No	7.70 (11.2)	1.50 (1.9)	1.60 (2.2)	2.90 (3.5)
	Yes	8.55 (11.2)	1.75 (2.1)	1.90 (2.5)	**3**.**30 **(**3**.**6**)*
	**Current Employment**				
	No	7.30 (9.8)	1.50 (2.1)	1.40 (2.3)	2.70 (3.0)
	Yes	**8**.**40 **(**12**.**5**)*	1.63 (1.9)	1.80 (2.3)	**3**.**20 **(**4**.**2**)*
	**Parity**				
	Nulliparous	7.20 (7.2)	1.60 (1.3)	1.50 (2.0)	2.90 (3.7)
	Multiparous	8.10 (12.5)	1.60 (2.4)	1.70 (2.4)	3.00 (3.5)

^a^Sample size for PBDE 153 drops by one subject as the analytical sample failed laboratory QA/QC.

^b^For samples with PBDE concentrations < LOD, the value of LOD/sqrt 2 was substituted.

*indicates Kruskal-Wallis significance at p < 0.1, ** indicates Kruskal-Wallis significance at p < 0.05.

Using generalized gamma or log logistic regression models, we identified unique predictor patterns for each PBDE congener and calculated the total variance explained by the combination of predictors (adjusted R^2^) (Table 4). Adjusted R^2^ ranged from 4.6% (PBDE-99) to 24.5% (PBDE-153).

**Table 4:** Ratio of geometric means derived from regression models for specific PBDE congeners measured in maternal blood collected upon delivery

	**PBDE 47**	**PBDE 99**	**PBDE 100**	**PBDE 153**
**# < LOD / Total observations**^**a**^	**3 / 311**	**50 / 313**	**28 / 316**	**5 / 304**
**Model distribution**	**General Gamma**	**Log logistic**	**Log logistic**	**General Gamma**
	**Ratio of geometric means (95% CI)**	**Ratio of geometric means (95% CI)**	**Ratio of geometric means (95% CI)**	**Ratio of geometric means (95% CI)**
**Race**/**ethnicity**				
Non Hispanic White	1			
African-American	1.47 (0.95, 2.29)^b^			
Hispanic	1.21 (0.92, 1.57)			
**Maternal education**				
Less than high school	1	1		1
High school or equivalent	1.76 (1.30, 2.37)*	1.39 (0.98, 1.97)^b^		1.21 (0.92, 1.60)
College	1.59 (1.20, 2.10)*	1.27 (0.93, 1.73)		1.42 (1.10, 1.83)**
Graduate	1.45 (0.99, 2.12)^b^	1.00 (0.69, 1.45)		0.95 (0.67, 1.34)
**Household income**				
< $25,000				1
$25,000 – 50,000				1.22 (0.93, 1.62)
>$50,000				1.47 (1.15, 1.87)**
**Pre**-**pregnancy BMI**				
Normal				1
Underweight				0.81 (0.57, 1.16)
Overweight				0.99 (0.81, 1.21)
Obese				0.65 (0.50, 0.85)**
**Solid Dairy** (**reference**:)				
< 5 servings per week				1
5-10 servings per week				1.19 (0.97, 1.47)
>10 servings per week				1.25 (1.01, 1.56)*
**Processed Meat**				
≤1 serving per week			1	1
Not reported			1.06 (0.79, 1.40)	1.14 (0.91, 1.44)
>1 servings per week			1.39 (1.10,1.76)**	1.53 (1.26, 1.86)**
**Household electronics**				
< 10 electronics	1	1	1	1
≥ 10 electronics	1.70 (1.35, 2.13)**	1.41 (1.12, 1.77)**	1.37 (1.08, 1.74)**	1.32 (1.07, 1.63)**
Generalized R^2^^c^	**12**.**2**%	**4**.**6**%	**5**.**7**%	**24**.**5**%

^a^Sample size for each congener changes due to missing covariates.

^b^0.05 < p < 0.09, *p < 0.05, ** p < 0.01.

^c^Generalized R2 indicates the proportion of variability in the data that is accounted for by the statistical model with multiple predictors.

## Discussion

Recent experimental data indicates PBDEs are developmental neurotoxicants and exposure during gestation is associated with adverse neurologic outcomes (2, 21). Understanding the predictors of exposure to PBDEs among pregnant women and women of childbearing age may help identify highly exposed subpopulations and help inform interventions to reduce exposure. This study examined body burdens of PBDEs and identified determinants of exposure among a sample of healthy pregnant women enrolled in a predominantly low-income, Hispanic cohort who provided maternal blood samples between years 2009 and 2010.

PBDE exposure is widespread in this cohort. At least one PBDE congener was detected in serum collected from each subject and 4 congeners (PBDE-47, -99, -100 and -153) were detected in nearly 100% of serum samples. Our analyses demonstrate unique demographic and dietary predictor patterns for serum PBDE-47, -99, -100 and -153. Race/ethnicity appeared predictive of PBDE body burden; levels of PBDE-153 were 20% higher among Non Hispanic Whites compared to Hispanics and 13% higher than African Americans. Though not significant, levels of PBDE-47 were 20% higher among African Americans compared to Non Hispanic Whites and Hispanics. Maternal education level is also associated with maternal PBDE body burden; levels of PBDE-47 were highest among subjects with a high school education compared to all other categories and levels of PBDE-153 were highest among college graduates compared to other categories. Consumption of solid dairy and consumption of processed meat were weakly associated with higher levels of all 4 PBDE congeners. Reporting of a greater number (≥ 10) of household electronics was significantly associated with higher levels of all 4 commonly detected PBDE congeners. More characteristics were associated with serum PBDE-153 than for any other congener.

### Levels and patterns of PBDE exposure

While PBDE exposure is widespread among this cohort, levels were lower than those reported in any other U.S. based cohort of pregnant women. Within the last decade, several studies have reported levels of PBDEs in blood samples collected during pregnancy or immediately following pregnancy [26-30], for review see [31]. Woodruff et al. (2012) reviewed Nurses Health And Nutrition Examination Survey (NHANES) data for chemicals present in samples collected from U.S. pregnant women between 2003/2004 [30]. Levels of PBDEs measured in NHANES women were 3–4 times higher than those measured in our cohort. Similarly, a recent study of predominantly Non-Hispanic Black pregnant women living in North Carolina between years 2008–2010 reported levels of PBDEs similar to those reported in NHANES and 3–4 times higher than our levels [28]. Levels of PBDEs measured in an ethnically diverse cohort of pregnant women living in California and sampled between 2008/2009 were among the highest reported in the country [32]. These levels were double the national values [29] and up to 6 times higher than those measured in our cohort. Herbstman et al. (2010), presented levels of PBDEs measured in cord blood samples collected from infants born in New York City in 2001 [33]. Maternal and fetal blood levels of PBDEs are highly correlated and a strong indication of PBDE exposure [34]. Cord blood PBDE levels in the Herbstman study were nearly two times higher than our reported maternal blood levels with the exception of PBDE-153, which was only 12% higher in the 2001 cohort than our cohort.

The generally lower levels of PBDEs detected in our cohort may be due to statewide legislation and abatement policies regarding the manufacture and use of PBDEs. Due to public concerns and economic reasons, Great Lakes Chemical Corporation, the only North American producer of PBDEs, voluntarily stopped producing octa-BDE and penta-BDE mixtures in December 2004 [35,36]. By 2006, New York State banned the manufacturing, processing, or distributing products penta- and octa-PBDEs and initiated a phase out of deca-PBDE by 2008 [37,38]. While California also banned penta- and octa-PBDEs by 2006 [39], the state California maintains the nations highest flammability standards which were enacted in the 1970’s [40]. These standards may explain the high levels of PBDEs measured in California residents.

While lower than most other U.S. cohorts, PBDE levels in this cohort were up to 8 times higher than those measured in the Netherlands and Sweden [31]. This is consistent with estimates that North Americans have the highest global body burden of PBDEs worldwide, averaging 20 times that of Europeans [41]. Lower levels in European countries may be due to earlier and stricter enactment of regulations in 2004 restricting the use of octa-and penta-congeners [41].

Though levels measured in our cohort were lower than other US cohorts and higher than international cohorts, the pattern of distribution of PBDE congeners is similar to other patterns reported for PBDEs measured in maternal and/or umbilical cord blood samples (reviewed in [31]). Similar to other studies, the distribution of PBDEs in our cohort is not normal, and approximately 5% of subjects have PBDE concentrations 50x times higher than the median [9,10,12,42]. Also consistent with the literature, PBDE-47, -99 and -100 are more highly correlated with each other than with PBDE-153 [43,44]. PBDE-47, -99 and -100 are all components of the penta-PBDE technical mixture.

Commercial penta-PBDE is most commonly used as a flame retardant in polyurethane foam [45]. The origin of these congeners in the environment is likely due to off gassing from products containing the technical penta-PBDE mixture such as upholstered couches, mattresses, mattress pads, and other foam items and from degradation pathways of deca-PBDE [46]. PBDE-47 may be the predominant congener due to its higher potential for bioaccumulation [47] or due to other unidentified sources of exposure [46]. PBDE-153 is a component of both penta-PBDE and octa-PBDE technical mixtures. Octa-PBDE mixtures are used most widely in plastics and textiles [48]. Notably, though PBDE-153 was widely detected in biological samples (reviewed in [31]), it contributes very little to the overall content of PBDEs in technical products; only 0.15% to 8.7% of the composition of octa-PBDE and 5.3- to 5.4% of penta-PBDE. In rodents, PBDE-153 is resistant to metabolism, which, if also applicable to humans may explain the proportionately higher PBDE-153 concentrations in human matrices compared to commercial mixtures [43,49].

### Predictors/sources of exposure

While PBDE exposure in the general population has been associated with the indoor environment and diet [10,12,16,50-53], few studies consider individual sources of exposure to PBDEs during pregnancy. In one recent study of a largely Mexican immigrant population of pregnant women living in California, PBDEs concentrations increased with increasing years residing in the U.S. and with the number of pieces of stuffed furniture in the home [28]. Herbstman (2007) [42] examined associations between PBDE levels in cord blood and potential predictors in 94 fetal cord serum samples. Predictors of cord blood PBDE-47 and -153 included younger maternal age and less weight gaining during pregnancy. In our study, maternal age was not associated with any PBDE congener and, while we do have information on weight gain during pregnancy, prepregnancy BMI was only weakly associated with higher levels of PBDE-153.

Two recent studies examined predictors of PBDEs among U.S. born girls [43,54]. Both studies found that social factors influenced children’s body burden levels. Lower socioeconomic status, assessed using educational level of the mother, was associated with higher PBDE levels in children. Rose et al. (2010) found that among their largely Hispanic and White population, Hispanic children had lower levels of higher brominated congeners (sum PBDE-197 thru -209) but no significant differences in race/ethnicity were observed among the lower brominated congeners (sum PBDE-28 thru -153). In the Windham study, Black girls had significantly higher levels of PBDEs compared to whites, Hispanics had intermediate values. Authors report that the reasons for racial and socioeconomic disparity in PBDE exposure are unknown but may reflect differences in exposure pathways, specifically housing factors or household furnishings.

Among our predominantly Hispanic cohort, we observed higher levels of PBDE-47 in African Americans compared to Hispanic and non-Hispanic white mothers, though differences were not significant. PBDE-153 levels were highest among non-Hispanic whites and lowest among Hispanics. We did not observe associations between income and PBDE levels in our cohort, though education level was predictive of PBDE congeners. PBDE-47 levels were highest among those mothers reporting a high school education compared to those with a less than high school education and those with higher levels of education (college or graduate school). PBDE-153 was highest among college graduates compared with those subject with lower education and those with higher education. The different trends observed between our cohort and the previously discussed cohorts may again be associated with the time of sample collection, the catchment areas of the enrollment and the different demographic distribution of the cohorts.

Food intake is an important source of exposure to PBDEs. PBDEs accumulate in lipid rich tissues. Food items like fish from high trophic levels and lipid-rich oils have been found to contain relatively high concentrations of PBDEs [55,56]. In addition, bioaccessibility of PBDEs is higher among fatty foods [57]. Among food items, fish, meats, and dairy products have the highest PBDE concentrations[2]. PBDEs are less commonly detected in fruits and vegetables [7], however contamination of produce is known to occur through the process of ‘hydro-cooling’ using plastic pallets coated with deca-PBDE [58]. A recent study quantified levels of PBDEs in commonly consumed foods in the United States and showed the highest levels of PBDEs in butter and fish, and the lowest detectable levels in other liquid dairy products (milk and yogurt) and vegetables [59]. Further, while fish tend to be more highly contaminated with PBDEs than meat and dairy, meat and dairy are more important sources of PBDE exposure in the United States [60,61]. Previous studies have attempted to quantify PBDE concentrations and dietary intake [50,62]. Modest associations have been reported between serum PBDEs and fish consumption among consumers of sport caught fish [63-65]. A recent study using NHANES data examined the association between food items from a 24-hour recall and a 1-year food frequency questionnaire and serum PBDE levels. Intake of poultry and red meat contributed significantly to PBDE body burden in the U.S. [66]. Recent market basket survey show contamination highest in fish, then meat and least in dairy products [50], but the average American has most dietary PBDE intake from meat, then dairy and fish [61].

In the current study, we examined associations between PBDE congeners and dietary consumption focusing on meat, poultry, dairy products, fish and shellfish. Women with higher levels of PBDE-153 and -100 were more likely to report consumption of processed meat. Women with higher levels of PBDE-153 were also more likely to report consumption of solid (high fat) dairy products such as cheese, cottage cheese and butter. We did not observe an association between consumption of fish, including fatty fish, and any PBDE congener.

A recent review investigated human internal and external exposure to PBDEs and suggested that while diet was long considered the most important source of human exposure to PBDEs, the unintentional ingestion of dust is at least of similar importance [55]. It is likely that PBDEs migrate out of household products including furniture and electronics and are sequestered in house dust. House dust then contributes to PBDE body burden either through oral intake of the dust or via dermal absorption [14]. In the current study, we queried women about the number of household electric appliances in the home (i.e., televisions, computers, microwave ovens). We observed higher levels of PBDE-47, -99, -100 and -153 among women who reported at least 10 household electronic and electrical appliances (including stereo equipment, televisions, toasters, microwaves, computers and/or printers). Based on the literature, we anticipated that household electronics would predict deca-PBDEs rather than penta-PBDEs. Deca-PBDE (PBDE-209) was measured in serum samples in our cohort and detected in less than 20% of subjects. We did not observe associations between PBDE-209 and household electronics (data not shown). Interestingly, the association between PBDE-153 and household electronics was stronger among Hispanics than whites or African Americans and strongest among those subjects in the lowest income category. The association between electronics and PBDE-47 was strongest among Whites and Hispanics and consistently strong across all income categories. It is possible that the types of electronics in the home may differ by race/ethnicity and income, thus influencing the exposure profile.

Our study has several limitations. With respect to questionnaire data, several recent studies suggest PBDE body burden differs by race/ethnicity, country of origin and years living in the U.S. Rose et al. reported that children of foreign-born mothers, 52% of whom were Hispanic, had significantly lower levels of higher brominated PBDE congeners than those whose mothers were born in the U.S. [67]. While we have a predominately Hispanic population, we do not have information on the country of birth (U.S. or foreign born) or the number of years living in the U.S.

In addition to dietary and consumer products, housing characteristics reflecting lifestyle are associated with PBDE exposure. One study found that children living in larger homes had significantly lower levels of PBDE-209; higher maternal education was correlated with larger homes [67]. Dust concentrations of PBDEs within larger homes may be lower since increasing square footage may dilute the PBDE contamination from a household source [17]. In the current study, we do not have information on housing quality or size.

With respect to environmental and biological monitoring data, we do not have environmental data on PBDEs concentrations in house dust or indoor air, nor do we have PBDE levels on the foods consumed by women. Further, we have no assessment of dermal absorption. Biological exposure data may not be directly correlated to source exposure data due to individual metabolism. In addition, we do not have measures of PBDE metabolites. In mice, PBDEs are metabolized in vivo to para- and ortho-hydroxylated metabolites (HO-PBDEs) [68]. It has been suggested that the para-HO-PBDE metabolites are the most abundant and potentially the most toxic metabolites due to hormonal activity [69]. While it is possible to measure para-hydroxylated metabolites in blood collected from pregnant women [29,70], metabolite biomarkers were not available for this study. Finally, with respect to questionnaire items querying participants about household items likely to contain PBDEs, we did not ask the age or condition of household items. It is possible that newly purchased items are a greater source of exposure to PBDEs due to increased off gassing. Alternatively, it is possible that older items may be a greater source of exposure due to breakdown of the construction materials. Our questionnaire items querying housing characteristics did not include information on number of bedrooms, square footage, or proxies for air exchange and ventilation. As air exchange rates and size of home may affect PBDE concentrations in the home [17], we are unable to assess this.

## Conclusions

While PBDE exposure appears widespread among this low income, multi-ethnic cohort of healthy pregnant women living in New York City, levels were low compared to other US populations of pregnant women. Lower levels in this cohort may be due to the timeliness of our sample collection, which occurred following considerable state-wide and nationwide legislation to reduce PBDEs. While recent policies and legislation to ban the manufacture and use of PBDEs appear to be effective at reducing exposure, our data confirm continued exposure through dietary and consumer products. These data underscore the need to continue regulatory screening and preventative measures to prevent similar bad actors from being introduced into commerce.

While our attempt to identify unique predictor patterns of PBDE body burden revealed several potential predictors such as intake of fatty foods (high fat dairy and processed meat), and number of household electronics, we did not observe a single predictor or a consistent pattern of predictors representing a significant source of exposure for any individual PBDE congener. Limiting intake of foods likely to contain PBDEs (processed meats and high fat dairy) and reducing the number of electronics in the home may help reduce exposure, however in our data there is little evidence to suggest that individual actions may considerably reduce PBDE exposures. Rather, our results reinforce the persistence of these compounds and highlight the need for continued legislation and regulatory screening before chemicals are introduced into commerce.

## Abbreviations

CDC: Centers for Disease Control and Prevention; IRB: Institutional Review Board; LOD: Limit of Detection; NHANES: National Health and Nutrition Examination Survey; PBDE: Polybrominated diphenyl ether; PCB: Polychlorinated biphenyl ether; POP: Persistent organic pollutant; QA/QC: Quality assurance/quality control.

## Competing interests

The authors confirm there are no competing financial interests in the collection, interpretation or presentation of this data. The views expressed in this document are solely those of the authors and do not necessarily represent those of the Centers for Disease Control and Prevention.

## Authors’ contributions

All authors of this research paper have directly participated in the planning, execution, or analysis of the study. All authors have read and approved the final version of this paper being submitted, and accept responsibility for the manuscript contents. MH participated in the design and coordination of the study, performed the statistical analysis and designed the tables and figures. SB coordinated the selection and enrollment of study subjects and managed the database. RJ performed the laboratory analyses. AS participated in the study design and development of the manuscript and supervised the laboratory analyses. XL participated in the study design and supervised the statistical analyses. RWhyatt participated in the conception of the study and development of the study design. RWapner participated in the conception of the study and development of the study design. PF, primary investigator, conceived of the study and participated in the design and coordination, helped with statistical analyses and with drafting the manuscript.
